# Hypertrophy-Reduced Autophagy Causes Cardiac Dysfunction by Directly Impacting Cardiomyocyte Contractility

**DOI:** 10.3390/cells10040805

**Published:** 2021-04-04

**Authors:** Christiane Ott, Tobias Jung, Sarah Brix, Cathleen John, Iris R. Betz, Anna Foryst-Ludwig, Stefanie Deubel, Wolfgang M. Kuebler, Tilman Grune, Ulrich Kintscher, Jana Grune

**Affiliations:** 1 Department of Molecular Toxicology, German Institute of Human Nutrition Potsdam-Rehbruecke, 14558 Nuthetal, Germany; Tobias.Jung@dife.de (T.J.); Cathleen.John@outlook.de (C.J.); Stefanie.Deubel@dife.de (S.D.); scientific.director@dife.de (T.G.); 2 DZHK (German Centre for Cardiovascular Research), Partner Site Berlin, 10785 Berlin, Germany; sbrix@gmx.de (S.B.); iris.betz@charite.de (I.R.B.); anna.foryst@charite.de (A.F.-L.); Wolfgang.Kuebler@charite.de (W.M.K.); ulrich.kintscher@charite.de (U.K.); jana.grune@charite.de (J.G.); 3 Center for Cardiovascular Research, Institute of Pharmacology, Charité-Universitaetsmedizin, 10115 Berlin, Germany; 4 Institute of Physiology, Charité-Universitaetsmedizin, 10115 Berlin, Germany; 5 German Center for Diabetes Research, 85764 München-Neuherberg, Germany; 6 Institute of Nutritional Science, University of Potsdam, 14558 Nuthetal, Germany; 7 Center for Systems Biology, Massachusetts General Hospital Research Institute, Harvard Medical School, Boston, MA 02114, USA

**Keywords:** rapamycin, neonatal cardiomyocyte contractility, TAC, hypertrophy, autophagy, siAtg5

## Abstract

Cardiac remodeling and contractile dysfunction are leading causes in hypertrophy-associated heart failure (HF), increasing with a population’s rising age. A hallmark of aged and diseased hearts is the accumulation of modified proteins caused by an impaired autophagy-lysosomal-pathway. Although, autophagy inducer rapamycin has been described to exert cardioprotective effects, it remains to be shown whether these effects can be attributed to improved cardiomyocyte autophagy and contractility. In vivo hypertrophy was induced by transverse aortic constriction (TAC), with mice receiving daily rapamycin injections beginning six weeks after surgery for four weeks. Echocardiographic analysis demonstrated TAC-induced HF and protein analyses showed abundance of modified proteins in TAC-hearts after 10 weeks, both reduced by rapamycin. In vitro, cardiomyocyte hypertrophy was mimicked by endothelin 1 (ET-1) and autophagy manipulated by silencing Atg5 in neonatal cardiomyocytes. ET-1 and siAtg5 decreased Atg5–Atg12 and LC3-II, increased natriuretic peptides, and decreased amplitude and early phase of contraction in cardiomyocytes, the latter two evaluated using ImageJ macro Myocyter recently developed by us. ET-1 further decreased cell contractility in control but not in siAtg5 cells. In conclusion, ET-1 decreased autophagy and cardiomyocyte contractility, in line with siAtg5-treated cells and the results of TAC-mice demonstrating a crucial role for autophagy in cardiomyocyte contractility and cardiac performance.

## 1. Introduction

Since the percentage of the elderly entering ≥65 years of age is rapidly increasing, age-related diseases, particularly cardiovascular diseases (CVDs) are the number one diseases contributing to mortality worldwide [1]. A better understanding of the underlying mechanisms for the development of CVDs is inevitable to prevent the progression of cardiac remodeling and failure. Structurally, pressure overload in the heart initially increases left ventricular mass and cell size to elevate force of contraction, which can progress to left ventricular hypertrophy [2], high serum levels of natriuretic peptides get released, brain natriuretic peptide (BNP) and atrial natriuretic peptide (ANP) [3], cardiomyocyte contractility declines and the formation of reactive oxygen species increases (ROS) [4], all of which resulting in heart failure (HF) [5,6,7]. Since cardiomyocytes are widely accepted as post-mitotic cells, functional and balanced proteostasis is essential to maintain cardiac homeostasis and performance. Degradation systems, like the ubiquitin-proteasomal system or autophagy-lysosomal pathway (ALP), avoid the accumulation of highly modified, cross-linked and defective proteins by intracellular turnover [8,9].

Macroautophagy (hereafter referred to as autophagy) is an evolutionally conserved, cell-autonomous process, delivering cytosolic material to the lysosomes for degradation by lysosomal hydrolases. Among several autophagy-related proteins, microtubule-associated proteins 1A/1B light chain 3B (LC3) and p62/SQSTM (p62) are widely used to estimate the autophagic flux [8,10]. A crucial regulator of autophagy is the mechanistic target of rapamycin (mTOR), suppressing early steps in autophagy [7]. Thereby, mTOR forms two distinct complexes, mTORC1 and mTORC2, with different sensitivities to rapamycin [11,12].

Inhibition of mTOR using rapamycin (RAPA) abolishes suppression of initial autophagy by mTORC1 activity and is often used to activate autophagy [13]. The effects of RAPA can be verified by reduced phosphorylation of mTORC1 substrate protein ribosomal protein S6 kinase beta-1 (p70S6K) at Thr389 [10,14]. Upon autophagy activation, non-lipidated LC3-I is ligated to phosphatidylethanolamine (PE) at the isolation membrane, forming LC3-II [15]. Lipidation of LC3-I to LC3-II is facilitated by Atg5–Atg12 conjugate [16]. Besides its role in elongation of the isolation membrane to the autophagosome, LC3-II recruits p62 which selectively recognizes polyubiquitinated proteins (K63-linked), tagged for lysosomal degradation [17,18].

Autophagy’s role in the failing heart remains elusive and is still a matter of debate. Whereas some studies reported that retrieval of autophagy protects against cardiac hypertrophy, other studies found that autophagy is implicated in the pathogenesis of load-induced HF [19,20]. In ischemia-reperfusion injury, autophagic effects have been reported as beneficial during ischemia through an AMP-activated protein kinase 1(AMPK)-dependent mechanism, but harmful during reperfusion through a Beclin-1 mechanism, in which Beclin-1 delays autophagosome-lysosome fusion, highlighting autophagy’s role as a double-edged sword in CVDs [7,21]. In addition, pressure overload-induced heart failure was shown to constitute myocardial ROS resulting in the accumulation of oxidatively modified proteins [22,23] contributing, at least in part, to declined cardiomyocyte contractility.

Due to the fact that autophagy’s role in the failing heart is still a matter of ongoing debate, we aimed to investigate the role of autophagy during cardiac dysfunction in TAC-hearts and on contractility in ET-1 treated cardiomyocytes. Therefore, we analyzed whether (a) hypertrophy-reduced cardiac function in TAC-hearts can be improved by RAPA-induced autophagy, (b) ET-1-mimicked hypertrophy can decrease cardiomyocyte contractility by reducing autophagy, and (c) whether autophagy can directly impair cardiomyocyte contractility. Here, we demonstrate beneficial effects of RAPA-induced autophagy on cardiac remodeling by a reduced accumulation of modified proteins in long-term pressure overload leading to improved cardiac performance. Analysis of autophagy on cardiomyocyte function in presence of endothelin 1 (ET-1) and RAPA revealed impaired autophagy and contractility by ET-1, attenuated by RAPA. Using siAtg5 in spontaneously beating neonatal cardiomyocytes, we were able to directly reduce contractility and increase natriuretic peptides. In addition, the effect of ET-1 was reinforced when added to scramble controls rather than siAtg5 treated cells, indicating that ET-1-reduced cardiomyocyte contractility is caused by additional reduction of autophagy, demonstrating a crucial role of autophagy in cardiomyocyte function.

## 2. Materials and Methods

### 2.1. Animals

All animal procedures were performed in accordance with the guidelines of the German Law on the Protection of Animals (*n* = 31). The experimental protocol was reviewed and approved by the local authorities (Landesamt für Gesundheit und Soziales, Berlin, Germany). Animals used in this study were kept under identical housing conditions (12 h light/dark cycle, standard diet ad libitum). Male C57BL/6J mice (8–9 weeks old; Janvier Labs, Le Genest-Saint-Isle, France) underwent TAC surgery to induce left ventricular (LV) HF, as reported previously [24,25]. Prior to TAC surgery, each mouse was weighed to precisely calculate pain and anesthesia medication depending on individual body weight. Mice were anesthetized by i.p., injection of ketamine/xylazine (100 mg/kg BW/d, 20 mg/kg BW/d) (both Sigma-Aldrich, Steinheim, Germany) diluted in a total volume of 200 µL saline. Analgesia during surgical intervention was delivered using a single i.p., injection of carprofen (5 mg/kg BW/d, Pfizer, Berlin, Germany) diluted in a total volume of 150 µL saline. After thoracotomy, aortic constriction was carried out by placing a silk suture around the aorta between right and left carotid arteries and a 26-gauge needle. SHAM-operated animals underwent the same procedure except of the aortic banding. The total duration of the surgical procedure took a maximum of 45 min. Additionally, animals had access to drinking water containing metamizole (4.8 mg/mL water) seven days post-surgery, to further facilitate analgesia. Echocardiography was performed 5 weeks post-surgery to validate presence of aortic stenosis in TAC mice (Supplementary Materials, Table S1) [24]. Six weeks post-surgery, SHAM (*n* = 13) and TAC animals (*n* = 18) were divided into four groups: (1) SHAM VEH (*n* = 7), (2) TAC VEH (*n* = 9), (3) SHAM RAPA (*n* = 6), (4) TAC RAPA (*n* = 9) (Supplemental Table S2) and treated with either vehicle (VEH: NaCl + 0.5% Tween) or RAPA (2.5 mg/kg BW/d) via daily i.p., injections for 28 days. One TAC-animal died in week 8 post-surgery in the TAC RAPA group, reducing the total count of this group to *n* = 8. Tail-cuff measurements [26] of systolic blood pressure were performed in week 9 post-surgery. All animals were handled and pre-trained prior to final blood pressure measurements, to reduce stress levels during final experiments. Final echocardiography was performed one day before dissecting mice via cervical dislocation in week 10 post-surgery, collecting blood and tissue for further ex vivo analysis. Isolated hearts were immediately frozen in liquid-nitrogen and stored at −80 °C.

### 2.2. Echocardiography

Mice were anesthetized using 3% isoflurane and fixed in a supine position on a 37 °C heated pad with temperature and ECG monitoring. Echocardiography was performed using a 30 MHz center frequency transducer (18–38 MHz, center transmit: 30 MHz, axial resolution: 50 μm) together with a Vevo^®^ 3100 high-resolution Imaging System (both FUJIFILM VisualSonics, Toronto, ON, Canada), as previously described [24,27]. After hair removal, pre-warmed ultrasound gel (Parker Laboratories Fairfield, New Jersey, USA) was applied on the chest. Isoflurane concentrations were adjusted to a minimum (1–2%) to achieve constant and comparable heart rates during examination. Image analyses were performed by a single observer using the dedicated software package VevoLAB Version 2.0 (FUJIFILM VisualSonics, Toronto, ON, Canada). For the analysis of LV function and dimension, we acquired B- and M-Mode images of the LV in its maximum dimension in parasternal long axis view. Furthermore, velocity profiles of the ascending and descending aorta were carried out using pulsed-wave Doppler mode. All acquired images were digitally stored in raw format for further offline-analyses. LV dimensions and function were calculated according to the manufacturer’s instructions. Gradient P assessing the degree of aortic stenosis was calculated from velocity parameters as described previously. We analyzed three independent M-Mode pictures of each individual animal and 3 cardiac cycles in each of these pictures (*n* = 9 values per animal, subsequently averaged).

### 2.3. Blood-Pressure Measurement

Mean systolic blood pressure (SBP) was measured 9 weeks after surgical intervention using a PowerLab 5/20 device coupled to a non-invasive blood pressure controller (both ADInstruments, Oxford, UK) as described previously. Awake mice were placed in a tail vein restrainer and a pressure cuff (MLT125/M, ADInstruments, Oxford, UK) was wrapped around the tail. The mean SBP was calculated from 6–9 measurements per individual animal.

### 2.4. Immunohistochemistry

Paraffin sections (2 µm) were processed for immunohistochemistry [28]. Sections were deparaffinized with Roti^®^-Histol (Carl Roth, Karlsruhe, Germany 6640) and hydrated by ethanol gradient (100–40%). H&E staining was performed by firstly adding hematoxylin solution (Sigma-Aldrich, GHS316) for 45 s followed by 10 s tap water and incubation of Eosin (Sigma-Aldrich, HT110232) for 1 min. After staining, samples were mounted with Entellan^®^ (VWR, Radnor, PA, USA 1079610500). Whole heart sections were scanned using MIRAX Scanner from Zeiss (Oberkochen, Germany). Quantification of stained area (cell size) was performed using software MIRAX Viewer and ZEN 2 (blue edition) both from Zeiss.

### 2.5. Lysosomal Activity (Cathepsin D&E Activity)

Heart tissue samples were homogenized with a tissue lyser (ball mill, Qiagen, Hilden, Germany) in 500 µL of 1 mM DTT at 30 Hz for 2 min. Afterwards, homogenates were shaken in a thermomixer for 1 h at 4 °C, followed by sonication on ice for 2 min at 50% amplitude and centrifugation at 14,000 rpm for 20 min.

Further, 10 µg of lysates were incubated with incubation buffer (containing 24 mM Cystein*HCL, 150 mM Na-Acetate, 3 mM EDTA Dihydrate, pH 4.0) for 10 min. To measure cathepsins D&E activity, fluorogenic peptide (Enzo, Loerrach, Germany) #BML-P145) was used as substrate, final concentration of 166 µM. Fluorescence by AMC-cleavage was measured in a black 96-well plate after 1h at 37 °C, using 360/460 nm, respectively. Enzymes activity was calculated using free 7-amino-4-methylcoumarin (AMC) as standard.

### 2.6. Immunoblot Analyses

Cardiac tissue and cells were homogenized in lysis buffer (10 mM TrisHCl pH 7.5 0.9% NP–40 0.1% SDS, 1 mM Pefablock) with protease inhibitors, for tissue followed by ball mill (2 min, 30 Hz). Tissue homogenates were cleared by centrifugation (15 min, 14,000× *g*). Protein determination was performed via Lowry assay (DC™ Protein Assay Biorad).

Proteins were loaded into 7.5–15% SDS/PAGE gels and transferred to either 0.42 µm (for the transfer from gels 7.5–12% and detection of high molecular weight proteins) or 0.2 µm (for 15% gels and better detection of low molecular weight proteins, e.g., LC3-I, LC3-II) nitrocellulose membranes (VWR, 10600001). Signals were developed with fluorescent secondary antibodies (Li-cor, Lincoln, NE, USA; 1:15,000) of the Infrared Odyssey Imaging System. The used primary and secondary antibodies are listed in Table S3.

### 2.7. Cardiomyocyte Culture and Treatment

Neonatal mice at 1–3 days of age (*n* = 6) were decapitated using surgical scissors and the heart was removed via sternotomy. Freshly obtained cardiac tissue was minced using a sterile scalpel and subsequently washed with Hanks’ Balanced Salt Solution (HBSS) before enzymatic digestion (Cardiomyocyte Isolation Kit (#88281, Thermo Scientific™, Schwerte, Germany)). Culture dishes were precoated with fibronectin (0.5% (*v*/*v*) (Merck, Darmstadt, Germany; #F1141) in 0.02% (*w*/*v*) gelatin (Merck; #G9391) for 1 h at 37 °C before seeding the cells. Isolated cardiomyocytes were suspended in Dulbecco’s Modified Eagle Medium (DMEM with 10% heat-inactivated FBS ((Merck; #F2442)), 1% pen/strep (Biochrom, Berlin, Germany; #A2212) and 1 µL/mL growth supplement contained in the kit. According manufactures instructions, cells were cultured for a period of 7 days before starting the experiments. Cells were treated with either 100 nM RAPA (Merck, R8781), 100 nM ET-1 (Merck, E7764), 7 nM lysosomal inhibitor concanamycin A (ConA, Merck, C9705) or the respective combinations for the indicated time points.

### 2.8. Real-Time qPCR

Gene expression analysis of whole heart samples (*n* = 4 per group) was performed by isolating total RNA with RNeasy Micro kit (Qiagen). Reverse transcription was carried out using M-MLV-reverse transcriptase, RNAsin and dNTPs (all Promega, Madison, WI, USA) according to manufacturer’s instructions. Quantitative RT-PCR reactions were done in presence of fluorescent dye SybrGreen (Life Sciences, ABI, Warrington, United Kingdom) using a Mx3000P qPCR System (Agilent, Santa Clara, CA, USA). Relative mRNA expression levels were calculated after normalization to reference murine gene β-2-microglobulin (B2M). For mRNA analysis, cells were either treated with 100 nM ET-1, 100 nM RAPA or RAPA + ET-1 for 6 h before extracting mRNA with beads according to manufactures instructions (Dynabeads mRNA direct purification kit, ThermoFisher scientific, 61012). Afterwards, mRNA was reverse transcribed to cDNA using SensiFast™ cDNA Synthesis kit (# 65054, Bioline, BioCat, Heidelberg, Germany)) following the manufacturer’s instructions. Real-time qPCR was performed using ™ DreamTaq™ Hot Start DNA Polymerase (15619374, Thermo Fisher Scientific, Schwerte, Germany) and SYBR GREEN (Thermo Fisher Scientific, 10668265). Primers were purchased from Sigma Aldrich and listed in Table S4.

### 2.9. Atg5 Silencing

Atg5 siRNA (s62451, silencerSelect Atg5, Thermo Fisher Scientific sense: 5′GCAUUAUCCAAUUGGUUUAtt-3′; antisense: UAAACCAAUUGGAUAAUGCca) and a non-specific scrambled siRNA (AM4611, Silencer Negative control, Thermo Fisher Scientific) were resuspended in nuclease-free water (final 5 nmol). Neonatal cells were seeded in 24-well plates (according the isolation protocol) and transfected, using 3 µL Lipofectamine^®^ RNAiMAX transfection reagent (13778030) mixed with 47 µL reduced serum medium (Opti-MEM^®^ 31985-062, Thermo Fisher Scientific). Further, 50 µL Opti-MEM^®^ were mixed either with 1 µL Atg5 siRNA(si) or scramble control (sc) (10 pmol). Diluted si/sc were added to Lipofectamine^®^ RNAiMAX (1:1) and incubated for 20 min at room temperature (RT). Afterwards 50 µL of si/sc-lipid complexes were replenished with 450 µL culture media (*w*/*o* pen/strep) for a final volume of 500 µL/RNA concentration 5 pmol. Transfection was performed over 48 h, while transfection of the siRNA-lipid complex had to be renewed after 24 h to guarantee a sufficient downregulation.

### 2.10. Measurement of Cardiomyocyte Contractility

To characterize effects of siAtg5, ET-1 and RAPA on cellular contractility, isolated cardiomyocytes were treated for 6h using the respective conditions. Non-electrically stimulated, spontaneously contracting cardiomyocytes were recorded (at least 20 cells (technical replicates) per animal and condition), using a commercially available smartphone attached to the ocular of a microscope. Cells were recorded at 40-fold magnification in the transmitted light mode. Images were converted into avi-files and analyzed by ImageJ software (version 1.52 b), using the Myocyter v1.3. macro, recently developed by our group [29]. By analyzing changes in pixel intensity in each video of recorded cardiomyocytes, Myocyter captures cardiomyocyte contractility. Using Myocyter, we calculated, among others, the relative amplitude of contraction [25], representing the normalized extent of cell shortening during contraction (mean amplitude), and contraction times (s) at thresholds 20% of the amplitude (CT20).

### 2.11. Statistics

Statistical analyses were performed either by two-way-ANOVA followed by Bonferroni’s multiple comparisons test, one-way-ANOVA followed by Tukey’s posttest, unpaired t-test (indicated with #) or one-sample t-test (indicated with a) after testing for normal distribution, using Shapiro–Wilk test. Data are presented as mean + SEM of biological replicates. A *p*-value of <0.05 was assumed as statistically significant. All analyses were performed using GraphPad Prism 8.1.2 (332) software.

## 3. Results

### 3.1. RAPA-Treatment Improves LV Dysfunction in TAC Mice

**Figure 1:**
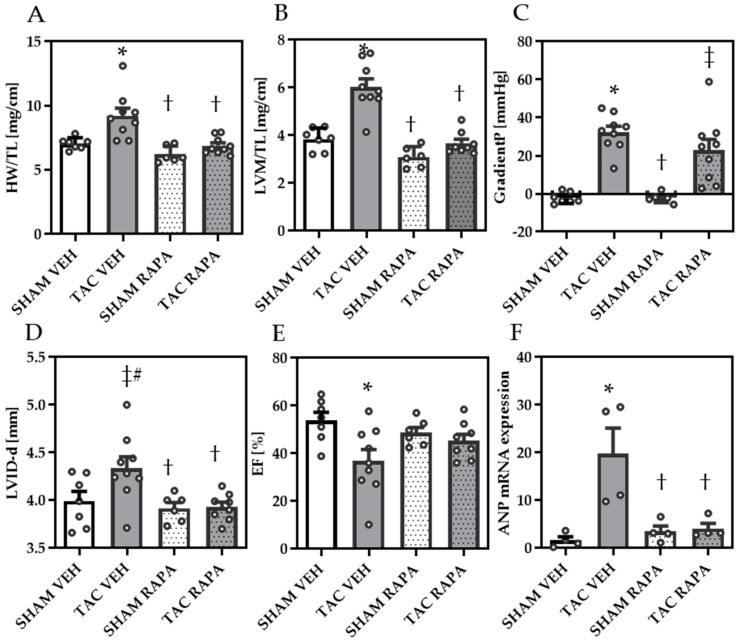
Pressure overload-induced heart failure is ameliorated by RAPA treatment. (**A**) Heart weight to tibia length (HW/TL). (**B**) Echocardiographic-assessed left ventricular mass matched to tibia length (LVM/TL). (**C**) Gradient P determining degree of aortic banding. (**D**) Echocardiographic-assessed diastolic left ventricular internal diameter (LVID-d). (**E**) Ejection fraction (EF). (**F**) Relative gene expression of atrial natriuretic peptide (ANP). Data are revealed 10 weeks post-surgery and presented as Mean + SEM of biological replicates. Statistical analyses were performed by either two-way-ANOVA followed by Bonferroni posttest or unpaired t- test (indicated with #). Significant differences are shown by *p* < 0.05, * vs. SHAM VEH, † vs. TAC VEH, ‡ vs. SHAM RAPA.

Ten weeks after surgery, heart weight to tibia length (HW/TL) and echocardiographic-assessed left ventricular mass (LVM) to tibia length ratios (LVM/TL) were significantly increased in TAC compared to SHAM VEH, demonstrating cardiac hypertrophy, while RAPA treatment showed a significant reduction of both in TAC mice (Figure 1A,B).

Doppler echocardiography revealed increased pressure gradients (GradientP) in TAC-mice, yet not significantly different between TAC VEH and TAC RAPA mice (*p* = 0.07) (Figure 1C, Table 1).

The mean diastolic LV internal diameter (LVID-d), indicating LV dilatation, tended to be increased in TAC compared to SHAM mice and was reduced in TAC RAPA compared to TAC VEH (Figure 1D). Global systolic function was decreased in TAC mice, demonstrated by reduced ejection fraction (EF), reduced fractional shortening and increased end-diastolic volume, all improved by RAPA treatment (Figure 1E, Table 1). Relative gene expression of atrial natriuretic peptide (ANP) was upregulated in TAC compared to SHAM VEH, which was again significantly reduced by RAPA treatment (Figure 1F).

### 3.2. RAPA Attenuated Cardiac Remodeling in Pressure-Overloaded Mice

**Figure 2:**
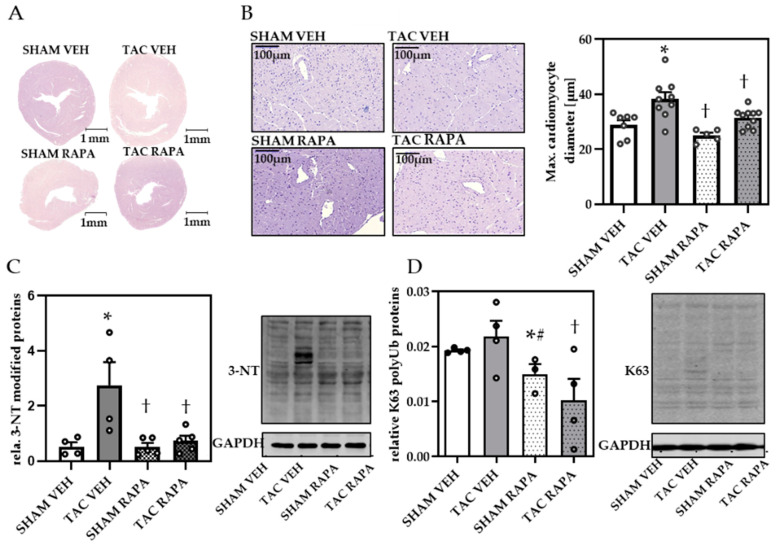
Rapamycin prevented TAC-induced cardiac remodeling in failing hearts. (**A**) H&E staining of cardiac cross-sections (magnification 1×, scale bar = 1 mm). (**B**) Representative images of H&E stained cardiomyocytes (magnification 20×, scale bar = 100 µm) and quantification of max. cardiomyocyte diameter. (**C**) Relative 3-nitrotyrosine (3-NT) quantification and representative immunoblot. (**D**) Relative K63-polyubiquitinated protein (K63) quantification and representative immunoblot. Immunoblot data are normalized to GAPDH and all data are presented as Mean + SEM of biological replicates. Statistical analyses were performed by two-way-ANOVA followed Tukey’s posttest or unpaired t- test (indicated with #). Statistically significant differences are shown by *p* < 0.05, * vs. SHAM VEH, † vs. TAC VEH.

To investigate the effects of RAPA on cardiac remodeling we initially compared cardiac cross sections and found increased cardiac cross-sections in TAC VEH compared to SHAM groups (Figure 2A).

Maximum cardiomyocyte diameters were increased in TAC compared to SHAM VEH, demonstrating cardiomyocyte hypertrophy, an effect rescued by RAPA treatment (Figure 2B). To examine whether pressure overload leads to impaired proteolysis we analyzed total 3-NT and K63 modified protein content, the latter marking proteins for selective autophagy. Immunoblot analysis displayed increased 3-NT (Figure 2C) and an upward trend of K63 (Figure 2D) modified proteins in TAC VEH, both significantly decreased by RAPA, indicating autophagy induction by RAPA treatment.

### 3.3. RAPA Treatment Leads to Improved Autophagy in Hypertrophic Conditions

**Figure 3:**
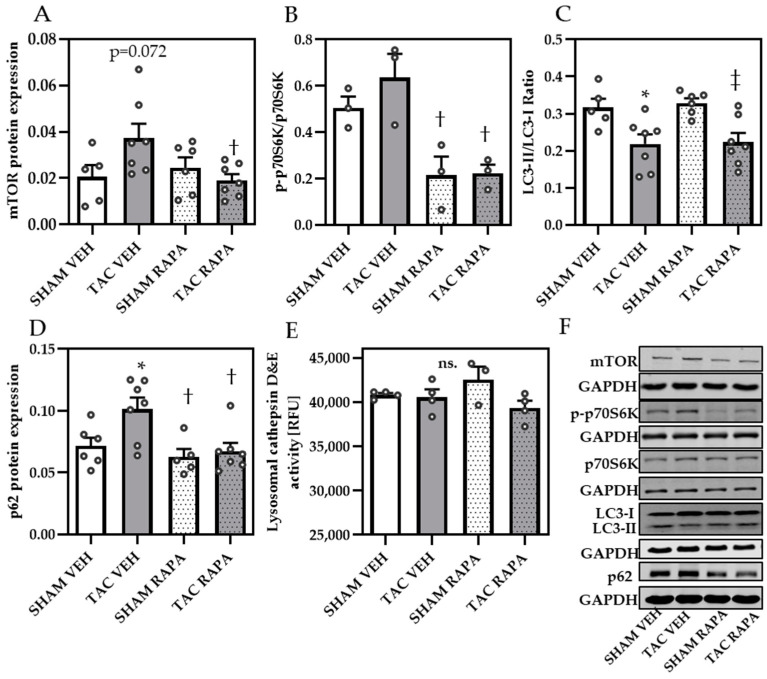
Rapamycin decreased mTOR- and increased autophagy-related proteins in TAC mice. Immunoblot analysis of: (**A**) mTOR, (**B**) p-p-70S6K/p-70S6K, (**C**) LC3-II/GAPDH to LC3-I/GAPDH ratio, (**D**) p62. (**E**) Maximal lysosomal cathepsin D&E activity. (**F**) Representative immunoblots of all mentioned proteins. Immunoblot data are normalized to GAPDH and all data are presented as Mean + SEM of biological replicates. Statistical analyses were performed by two-way-ANOVA followed by Tukey’s posttest. Statistically significant differences are shown by *p* < 0.05, * vs. SHAM VEH, † vs. TAC VEH and ‡ vs. SHAM RAPA, ns = not significant.

To assess whether RAPA-induced autophagy plays a role in preventing cardiac remodeling, we analyzed autophagy-related proteins in cardiac samples from all groups. Successful RAPA treatment and associated increased autophagy, was proven by diminished mTOR protein expression (Figure 3A,F) and suppressed phosphorylation status of mTORC1 target protein p70S6K (determined by ratio p-p70S6K/GAPDH to p70S6K/GAPDH) (Figure 3B,F) and mTORC2 substrate Akt-Ser 743 (Figure S1).

Analyzing the LC3-II/GAPDH to LC3-I/GAPDH ratio, we obtained significantly reduced values in TAC VEH compared to SHAM groups, however not affected by RAPA treatment (Figure 3C,F). We additionally quantified autophagic substrate p62 and found increased protein levels in TAC VEH compared to SHAM VEH, which were attenuated by RAPA (Figure 3D,F), indicating an increased p62 turnover by induced autophagy. In contrast, maximum lysosome cathepsin D&E activity was unchanged between all groups (Figure 3E), supporting the assumption that not lysosomal activity but altered autophagy contributes to an accumulation of modified proteins and cardiac remodeling in the heart.

**Figure 4:**
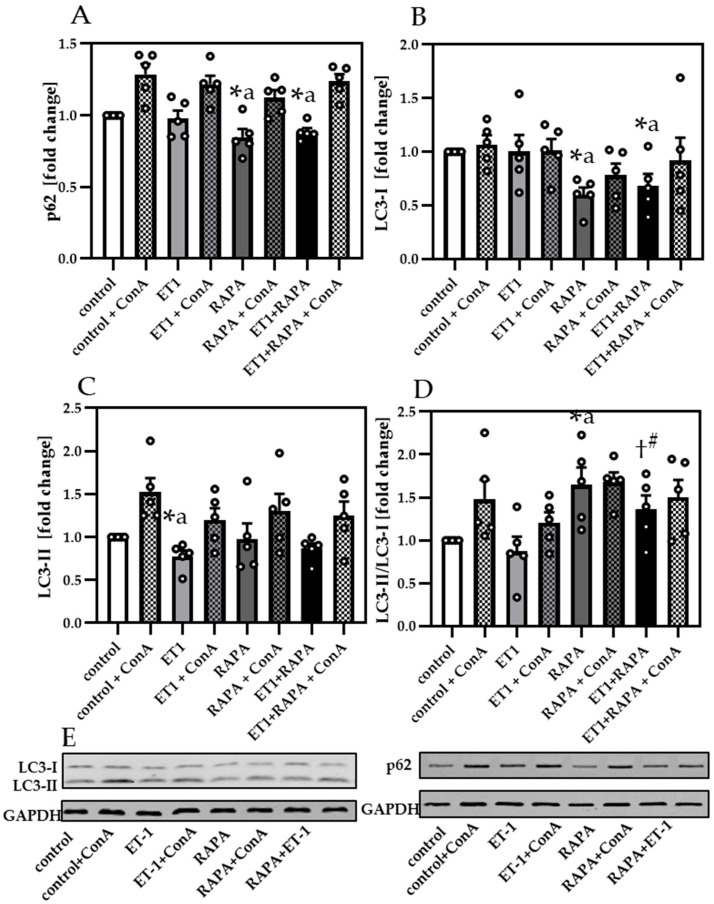
Endothelin-1 induced hypertrophy reduced autophagy in neonatal cardiomyocytes. (**A**) Immunoblot analysis of p62 and (**B**) LC3-I and (**C**) LC3-II. (**D**) Analysis of LC3-II/GAPDH to LC3-I/GAPDH. (**E**) Representative immunoblots of LC3-I, LC3-II, p62 and their respective GAPDH control. Data are presented as fold change (Mean + SEM) of biological replicates and statistical analyses were performed by unpaired *t*-test (indicated with #) or one-sample t-test (indicated with a), comparing two treatments directly. Statistically significant differences are shown by *p* < 0.05, * vs. control and † vs. ET-1.

We next aimed to investigate whether hypertrophy-reduced autophagy directly affects myocyte contractility. To address this question, we expanded our studies to an in vitro approach using isolated neonatal cardiomyocytes treated with ET-1 to mimic hypertrophic conditions. ET-1 associated hypertrophy was corroborated by increased BNP mRNA expression, which was significantly inhibited by additional RAPA treatment (Figure S2), in line with our findings in cardiac samples from pressure overloaded mice. Protein levels of p62 (Figure 4A,E) were unchanged by ET-1 but significantly decreased by RAPA treatment compared to control or ET-1, suggesting the activation of autophagy.

Analyzing LC3-I (Figure 4B,E) revealed reduced LC3-I expression by RAPA compared to control, while LC3-II was unchanged (Figure 4C,E). When comparing ET-1 to control we detected decreased LC3-II level. For both LC3 proteins, we observed an increased formation of LC3-II by LC3-I due to RAPA, compared to control or ET-1. Lipidation rate of LC3 was also demonstrated by the normalized ratio of LC3-II/LC3-I (Figure 4D). Normalized LC3-II/LC3-I ratios were unchanged by ET-1 compared to control, while RAPA increased the LC3-II/LC3-I formation in RAPA + ET-1, when compared to ET-1. Analyzing the LC3-II/LC3-I ratio in the absence or presence of ConA, we measured reduced levels in ET-1 samples (*p* = 0.06), indicating that ET-1 not only impairs LC3-II formation, but also the autophagic flux.

### 3.4. Impaired Autophagy Led to Decreased Cardiomyocyte Contractility

**Figure 5:**
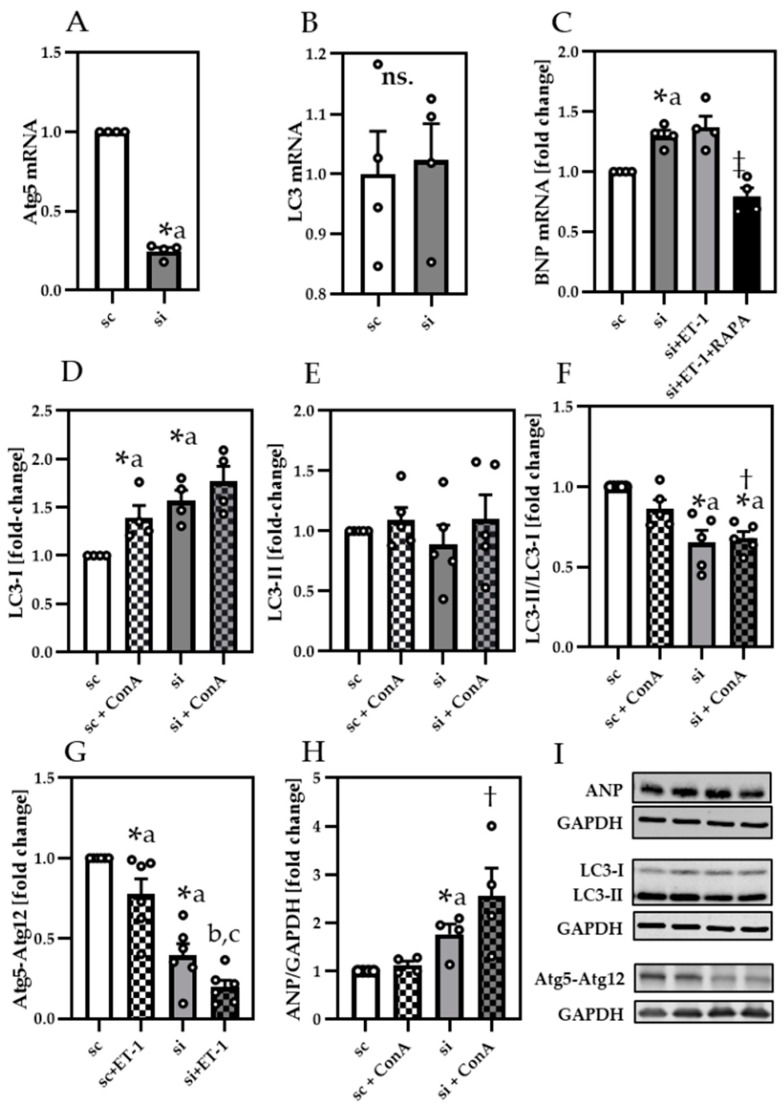
Atg5 silencing increased ANP and decreased LC3-II lipidation (**A**) Relative gene expression of Atg5, (**B**) LC3 and (**C**) BNP. Protein analysis of (**D**) LC3-I, (**E**) LC3-II, (**F**) LC3-II/GAPDH to LC3-I/GAPDH (**G**) Atg5-Atg12 conjugate and (**H**) atrial natriuretic peptide (ANP) in scramble control (sc) and siAtg5 (si) treated neonatal cardiomyocytes. (**I**) Representative immunoblots of ANP, LC3-I, LC3-II, Atg5-Atg12 conjugate and their respective GAPDH control. Data are presented as fold change of sc and shown as Mean + SEM of biological replicates. Statistical analyses were performed by unpaired t-test or one-sample t-test (indicated with a), comparing two treatments directly. Statistically significant differences were shown by *p* < 0.05, * vs. sc, ‡ vs. si + ET-1, † vs. sc + ConA, b vs. sc + ET-1 and c vs. si. ns = not significant.

To proof that insufficient autophagy may be linked to impaired contractile function, we silenced Atg5 in neonatal cardiomyocytes by using siRNA (Figure 5). While siAtg5 decreased Atg5 mRNA expression (Figure 5A), LC3 mRNA levels remained unchanged (Figure 5B).

In line with our results from pressure overload mice and ET-1 treated cardiomyocytes, Atg5-silenced (si) cells exhibited a prominent increase of BNP mRNA (Figure 5C), attenuated by RAPA. Since Atg5–Atg12 conjugate is known to contribute to LC3 lipidation, we additionally analyzed LC3-I/GAPDH (Figure 5D,I), LC3-II/GAPDH (Figure 5E,I) and LC3-II/LC3-I ratio (Figure 5F) in Atg5-silenced cells. While LC3-I was significantly increased in siAtg5 cells, LC3-II was unchanged, indicating less formation of LC3-II through reduced Atg5–Atg12 conjugate formation, in line with ET-1 (Figure 5G,I). Further, since LC3 mRNA expression was unchanged, less lipidation of LC3-I could cause the higher LC3-I levels in siAtg5-treated cells. Analyzing normalized LC3-II/LC3-I ratio we revealed significantly reduced values by siAtg5, confirming reduced LC3-II formation. Finally, ANP protein levels (Figure 5H,I) were upregulated by siAtg5, in line with BNP in ET-1 (Figure S2) suggesting that both can decrease cardiomyocyte function by impairing autophagy.

**Figure 6:**
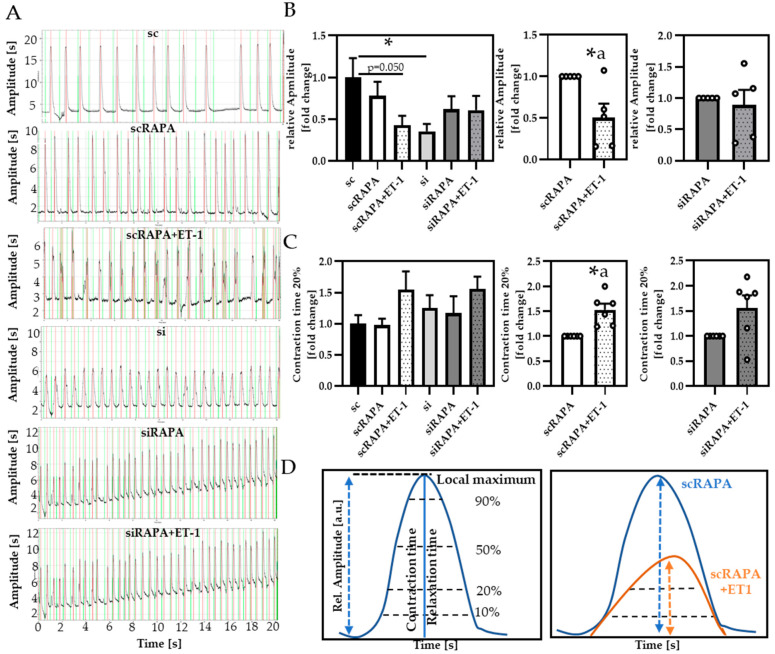
Atg5 siRNA and ET-1 decreased cardiomyocyte contractility by downregulation of autophagy. (**A**) Representative contraction profiles of the respective conditions, red local maxima, green local minima. (**B**) Relative amplitude of contraction and (**C**) Contraction time 20% (CT20). (**D**) Illustration of movement parameters analyzed in spontaneously contracting neonatal cardiomyocytes, using Myocyter. All data are presented as Mean + SEM of biological replicates and statistical analyses were performed be either one-way-ANOVA followed by Tukey’s posttest or One-sample *t*-test (indicated with a). Statistically significant differences are shown by *p* < 0.05, * vs. sc or scRAPA and *p* = 0.050 comparing sc vs. scRAPA + ET-1, using Unpaired *t*-test.

To verify whether impaired autophagy directly impacts cardiomyocyte contractility, we analyzed spontaneously contracting cardiomyocytes in respective conditions (Figure 6).

By recording beating cardiomyocytes and analyzing their motion parameters via the ImageJ macro Myocyter, we obtained contraction profiles for individual cells measured, exemplarily represented in Figure 6A. The Myocyter macro, recently developed by our group, enables detailed profiling of cardiomyocyte contractility (described in more detail in [29]) (Figure 6D). In our settings, we found a reduction of the relative amplitude of contraction in ET-1-treated cells compared to controls (Figure 6B). This effect was reinforced observed in scramble controls compared to siAtg5-treated cells, suggesting that ET-1-induced reduction of contractility is based on impaired autophagy. Analyzing the contraction time threshold 20% (CT20), calculated from the difference between minimum and maximum of cell shortening at certain thresholds, e.g., 20%, enables to distinguish whether amplitude of contraction is shifted or delayed and which phase of the cardiac cycle (contraction vs. relaxation) might be affected by the treatments (Figure 6D, left panel). ET-1 treatment increased CT20 compared to scramble controls, delaying the early contraction phase (Figure 6C), an effect less prominent in siAtg5-treated cells, leading to the assumption that ET-1 may directly impact the contractile function of cardiomyocytes via impaired autophagy. The relaxation phase was not affected by the treatments (data not shown).

## 4. Discussion

In the present study, we were able to show that impaired autophagy in hypertrophic conditions, is associated with reduced cardiac function by directly affecting contractility of hypertrophic cardiomyocytes.

Although autophagy can act as a double-edged sword in cardiac pathologies [5,21,30], basal protein turnover via autophagy is essential for adequate cardiomyocyte performance [31]. Disturbed protein turnover supports aggregation of modified, defective proteins, impacting appropriate cell function, particularly in post-mitotic cells (reviewed in [32]). Insufficient autophagy is a key event in the development of age-related diseases [33], e.g., cardiac hypertrophy and HF [34]. An anti-remodeling effect of RAPA was previously demonstrated by Bishu et al. in TAC mice receiving RAPA for 2 weeks [35]. In addition, our data complement positive effects of RAPA on cardiac function and autophagy, while lysosomal activity seems to be unaffected.

A study of Sala-Mercado et al. also demonstrated, that inhibition of initial autophagy, using a cell-permeable dominant negative Atg5 mutant, abolished cardioprotective effects of autophagy-inducing compound chloramphenicol, while blocking of lysosomal degradation with chloroquine did not disturb chloramphenicol-mediated cardioprotection, suggesting that sequestration of cytosolic material is more essential than liberation of breakdown-products [36]. In line with the literature, our data confirm that not lysosomal activity, but defective autophagy seems to provoke cardiac remodeling in TAC mice. Regarding TAC, studies described a distinct increase in the number of autophagosomes, peaking at 48h and persisted for at least three weeks post-TAC. Time course analysis showed a transiently activated autophagy immediately after TAC surgery which is downregulated five days after TAC [37].

Besides an upregulation of autophagosomes in response to pressure overload, autophagosome abundance can also reflect impaired autophagy, further increasing oxidative stress and oxidatively modified proteins. Elevated nitrated proteins were previously observed in mice with abdominal aortic banding [38], coinciding with the results from our study. In accordance with the literature [39,40,41], increased K63 and p62 protein level further indicate a reduced protein sequestration and turnover in TAC mice, improved by RAPA-induced autophagy. Given the fact that autophagy participates in reducing cellular mass and content, which is suppressed by mTOR activation, autophagy is generally suggested to be anti-hypertrophic. However, in conditions of energy restriction, it is conceivable that autophagy could contribute to the destruction of cells, that are incapable to counterbalance catabolism with anabolism [7]. Previous studies have already shown that late-life RAPA treatment significantly improved age-associated decreased systolic function in C57/BL6J mice [42] as well as short-term RAPA treatment did in middle-aged dogs [43].

Our finding, that ET-1 reduced the contraction amplitude in neonatal cardiomyocytes is in line with the literature, demonstrating involvement of endothelin receptor ETA [44]. Moreover, a study from Ceylan-Isik et al. showed that ET-1 triggers decreased autophagy, increased hypertrophic markers and impaired fractional and cardiomyocyte peak shortening, reversed by cardiomyocyte-specific ETA receptor antagonist BQ123 and RAPA-induced downregulation of ETA receptor [45]. Even if they showed that ET-1 decreased autophagy and contractility, a direct proof of autophagy-reduced contractility was missing and is demonstrated within the present study. Apart from mTOR-dependent autophagy, a multitude of signaling pathways can be addressed by RAPA, such as Nrf2/Keap1 [46], Nf-κB [47], or Jak2-STAT3 [48], in the heart, but also in the entire organism.

In line with the findings of Nakai et al., reporting that cardiac-specific deletion of Atg5 resulted in left ventricular dilatation, cardiac hypertrophy and contractile dysfunction in the heart [5], we demonstrated that Atg5 silencing directly impaired cardiomyocyte contractility. This is in line with data from Atg5 silenced and LPS-challenged cardiomyocytes, showing more apoptosis and decreased cardiomyocyte contractility compared to scramble LPS [49], indicating that Atg5 could impact cardiomyocyte contractility. Results presented herein prove that myocytes treated with siAtg5 provoke reduced cell shortening and increased natriuretic peptide expression. Further, we provide evidence that ET-1 may affect cardiomyocyte contractility via downregulation of autophagy. Since it was demonstrated that autophagosome formation was inhibited by modulated intracellular calcium homeostasis [50] and that ET-1 can activate calcium mobilization pathways [51], measuring free calcium and analyzing calcium signaling after treatment with hypertrophic stimuli in isolated cardiomyocytes might be a crucial future approach to further study hypertrophy-reduced autophagy.

## 5. Conclusions

In the present study, we demonstrate maintained LV function in vivo after RAPA treatment in hypertrophic conditions, which might be facilitated by enhanced cardiomyocyte contractility due to improved autophagy. Our data set provides important insights into the role of autophagy as a crucial mechanism underlying cardiomyocyte contractility in hypertrophic conditions. Beyond inducing autophagy, RAPA may also impact several other pathways. Hence, specific targeting of autophagic signaling pathways in aged and diseased cardiac tissue will be a crucial next step. Further, cytosolic calcium levels are strongly correlated to autophagosome formation and cardiac hypertrophy, hence future studies on calcium signaling and transcriptional regulation of autophagy-related proteins might be promising in understanding hypertrophy-reduced autophagy in diseased and aged hearts.

## Figures and Tables

**Table 1:** Echocardiography 10 weeks after intervention of heart failure cohort.

VEH			RAPA	
Cardiac Function and Dimension	SHAM	TAC	SHAM	TAC
No. of animals	7	9	6	8
HR (bpm)	489 ± 22.2	512.5 ± 16.5	536.2 ± 13.3	529.6 ± 19.5
LVM (mg)	66.9 ± 7.9	105.2 ± 7.4 *	52.5 ± 6.1 †	64.3 ± 3.7 †
LVID-d (mm)	4± ± 0.1	4.3 ± 0.1	3.9 ± 0.1b	3.9 ± 0.1 †
LVID-s (mm)	2.9 ± 0.2	3.6 ± 0.2 *	3.0 ± 0.1	3.1 ± 0.1
LVAW-d (mm)	0.6 ± 0.04	0.8 ± 0.03 *	0.5 ± 0.02 †	0.6 ± 0.03 †
LVAW-s (mm)	0.7 ± 0.1	1 ± 0.04 *	0.7 ± 0.02 †	0.8 ± 0.02 †
LVPW-d (mm)	0.6 ± 0.02	0.7 ± 0.2 *	0.5 ± 0.02*, †	0.6 ± 0.1 †
LVPW-s (mm)	0.8 ± 0.03	0.9 ± 0.03	0.7 ± 0.03 †	0.8 ± 0.03
EDV (µL)	70 ± 4.4	85.5 ± 5.5 *	66.8 ± 2.8 †	67.2 ± 2.1 †
ESV (µL)	33.2 ± 4.1	55.7 ± 7.6 *	34.5 ± 2.9	36.9 ± 2.3
SV (µL)	36.9 ± 1.2	29.8 ± 3.1	32.3 ± 1.5	30.3 ± 1.9
CO (mL/min)	18.1 ± 1.1	15.1 ± 1.5	17.4 ± 0.9	15.9 ± 0.9
EF (%)	53.8 ± 3.1	36.9 ± 4.8 *	48.7 ± 2.9	45.3 ± 2.7
FS (%)	27.7 ± 1.9	18 ± 2.5 *	24.2 ± 0.8	22.3 ± 1.6
Aortic vel. desc (mm/s)	1199 ± 72	1268 ± 139	1143 ± 100	1097 ± 105
Aortic vel. asc (mm/s)	−946 ± 74	−3107 ± 142 *	−887 ± 62 †	−2543 ± 257 *, ‡
Aortic Peak Pressure (mmHg)	3.7 ± 0.6	39.3 ± 3.4 *	3.2 ± 0.5 †	28 ± 5.3 *, ‡
Pressure Gradient P (mmHg)	−2.2 ± 1.1	32.2 ± 3.2 *	−2.2 ± 1 †	22.8 ± 5.8 *, ‡

Mean ± SEM of biological replicates. Two-Way-ANOVA followed by Bonferroni’s-posttest. *p* < 0.05. * vs. SHAM VEH-Group, † vs. TAC VEH-group, ‡ vs. SHAM RAPA-group. HR = heart rate; LVM = left ventricular mass; LVID-d = left ventricular internal diameter, diastolic; LVID-s = left ventricular internal diameter, systolic; LVAW-d = left ventricular anterior wall, diastolic; LVAW-s = left ventricular anterior wall, systolic; LVPW-d = left ventricular posterior wall, diastolic; LVPW-s = left ventricular posterior wall, systolic; ESV = end-systolic volume; EDV = end-diastolic volume; SV = stroke volume; CO = cardiac output; EF = ejection fraction; FS = fractional shortening; Ao. Peak Vel. Desc = aortic peak velocity, descendic; aortic peak velocity, ascendic.

## Data Availability

Experimental data used to support the findings of this study are available from the corresponding author upon request.

## Supplementary Materials

The following are available online at https://www.mdpi.com/article/10.3390/cells10040805/s1, Figure S1: Detection of Akt-Ser 473 in TAC heart tissue, Figure S2: BNP mRNA expression, Table S1: Echocardiography 5 Weeks after intervention (before start of treatment), Table S2: Gravimetric data 10 weeks after intervention of heart failure cohort, Table S3: Antibody list, Table S4: Primer sequences.
