# Supplementary material for: Hypertrophy-Reduced Autophagy Causes Cardiac Dysfunction by Directly Impacting Cardiomyocyte Contractility
Source: Cells. 2021 Apr 4;10(4):805. doi: 10.3390/cells10040805 (PMC8065800; doi:10.3390/cells10040805)
Supplement: Supplementary file 1 [file cells-10-00805-s001.pdf]

SUPPLEMENTAL MATERIAL

Hypertrophy-reduced autophagy causes cardiac dysfunction by directly impacting cardiomyocyte contractility

Christiane Ott, PhD*, Tobias Jung, PhD, Sarah Brix, PhD, Cathleen John, PhD, Iris R. Betz, Anna-Foryst-Ludwig, PhD, Stefanie Deubel, Wolfgang M. Kuebler, Prof., Tilman Grune, Prof., Ulrich Kintscher, Prof., Jana Grune, PhD

Expanded materials & methods

- **Supplemental Tables: 1-4**
- **Supplemental Figure 1-2**

Supplemental Table 1: Echocardiography 5 Weeks after intervention (before start of treatment)

	**VEH**		**RAPA**	
	**SHAM**	**TAC**	**SHAM**	**TAC**
No. of animals	7	9	6	9
Pulse-Wave Doppler Analysis				
Aortic Vel. desc [mm/s]	-923±86	-2766±180*	-938±189†	-2620±175*, ‡
Aortic Vel. asc [mm/s]	1135±114	1332±83	1160±145	1285±99
Aortic Peak Pressure [mmHg]	3.6±0.7	31.5±3.8*	4.2±1.9†	28.4±4.1*, ‡
Pressure Gradient P [mmHg]	-1.9±1.3	24.2±3.7*	-1.6±2.4†	21.5±4.6*, ‡

Mean±SEM. Two-Way-ANOVA followed by Bonferroni's-post test.p<0.05, * vs. SHAM VEH-Group, †vs. TAC VEH-group, ‡vs. SHAM RAPA-group. (First data analyses on SHAM/VEH and TAC/VEH groups were already published by us in (Grune et al., 2018 Cardiovasc Ultrasound) (PMID: 29966517).

Supplemental Table 2: Gravimetric data 10 weeks after intervention of heart failure cohort

**1.**	**VEH**		**RAPA**	
	**SHAM**	**TAC**	**SHAM**	**TAC**
No. of animals	7	9	6	8
**Physiological Data**				
BW week 5[a] (g)	26.8 + 0.2	27.5+0.3	26.4+0.4	26.8+0.4
BW week 10 (g)	28.0±0.3	28.9±0.3	26.6±0.4†	27.0±0.5†
**Necropsy Data**				
HW (mg)	123.0±8.3	160.3±29.1*	105.7±9.9†	120.7±11.7†
HW/BW (mg/g)	4.4±0.3	5.7±1*	4.0±0.3†	4.5±0.4†
LW (g)	193.6±38.3	178.4±44.2	189.3±40.1	194.6±25.3
LW/BW (mg)	6.9±1.4	6.4±1.6	7.1±1.4	7.2±0.9
Spleen (g)	74.3±22.3	90±17.8	65±18.7	73.3±20
Spleen/BW (mg/g)	2.7±0.8	3.2±0.6	2.4±0.7	2.7±0.8
**Blood Sample Analysis**				
Glucose (mg/dl)	192.7±24.7	192.2±40.6	176.8±39.9	174.3±55.5
**Tail Cuff Measurement**				
SBP (mmHg)	117.4±10.8	116.6±4.2	116.0±10.7	120.1±8.1

Mean±SEM. Two-Way-ANOVA followed by Bonferroni's-posttest $p<0.05$., * vs. SHAM VEH-Group. † vs. TAC VEH-group. BW=Body weight; HW=Heart weight; LW=Lung weight; SBP=Systolic blood pressure, [a] BW before intervention.

Supplemental Table 3: Antibody list

Primary antibody	Company/ article number
Anti-3-Nitrotyrosine antibody, mouse	Abcam, ab110282
Anti-SQSTM1 / p62 antibody mouse	Abcam, ab56416
Anti-LC3A/B rabbit (D3U4C)	Cell Signaling, #12741
Anti-mTOR (7C10) rabbit	Cell Signaling #2983S
Anti-Phospho-p70 S6 kinase (Thr389) (108D2) rabbit	Cell Signaling, #9234
Anti-p70 S6 Kinase (49D7) rabbit	Cell Signaling, #2708
Anti-Atg5 antibody, mouse (detecting Atg5 and Atg5-Atg12 conjugate	NanoTools ATG5-7C6
Anti-ANP, rabbit	Novus Biologicals #NBP2-14872
Anti-Lys63-linkage specific polyubiquitin antibody, rabbit	Cell signaling, #5621
Anti-GAPDH rabbit	Abcam, ab37168
Anti-GAPDH mouse	Abcam, ab8245
Anti-p-Akt (Ser 473), rabbit	Cell Signaling, #9271
Anti-Akt (Pan), rabbit	Cell Signaling, #4691
**Secondary antibody**	
IRDye® 800CW Donkey anti-mouse	Licor, 926-32212
IRDye® 800CW Goat anti-rabbit	Licor, 926-32211
IRDye® 680LT Donkey anti-mouse	Licor, 926-68022
IRDye® 680LT Goat anti-rabbit	Licor, 926-68021

Supplemental Table 4: Primer sequences.

		Forward Primer 5′-3′	Reverse Primer 5′-3′
RefSeq	*Mus Musculus*		
NM_009735.3	mmB2M	CTGCTACGTAACACAGTTCCACCC	CATGATGCTTGATCACATGTCTCG
NM_008725.3	mmANP	AGGAGAAGATGCCGGTAGAAGA	GCTTCCTCAGTCTGCTCACTCA
NM_008726.6	mmBNP	CACCGCTGGGAGGTCACT	GTGAGGCCTTGGTCCTTCAA
NM_025735.3	mmLC3	GACCAGCACCCCAGTAAGAT	TGGGACCAGAAACTTGGTCT
NM_008084.3	mmGAPDH	GGGTGTGAACCACGAGAAAT	GTCTTCTGGGTGGCAGTGAT
NM_013556.2	mmHrpt1	GCAGTCCCAGCGTCGTG	GGCCTCCCATCTCCTTCAT
NM_009438.5	mmRpl13a	GTTCGGCTGAAGCCTACCAG	TTCCGTAACCTCAAGATCTGCT

B2M=β-2-microglobulin, ANP= natriuretic peptide A, BNP=natriuretic peptide B. LC3 = Microtubule-associated proteins 1A/1B light chain 3B normalized to GAPDH = Glycerinaldehyd-3-phosphat-Dehydrogenase, Hrpt1= Hypoxanthine-guanine phosphoribosyltransferase, Rpl13a = Ribosomal Protein L13a

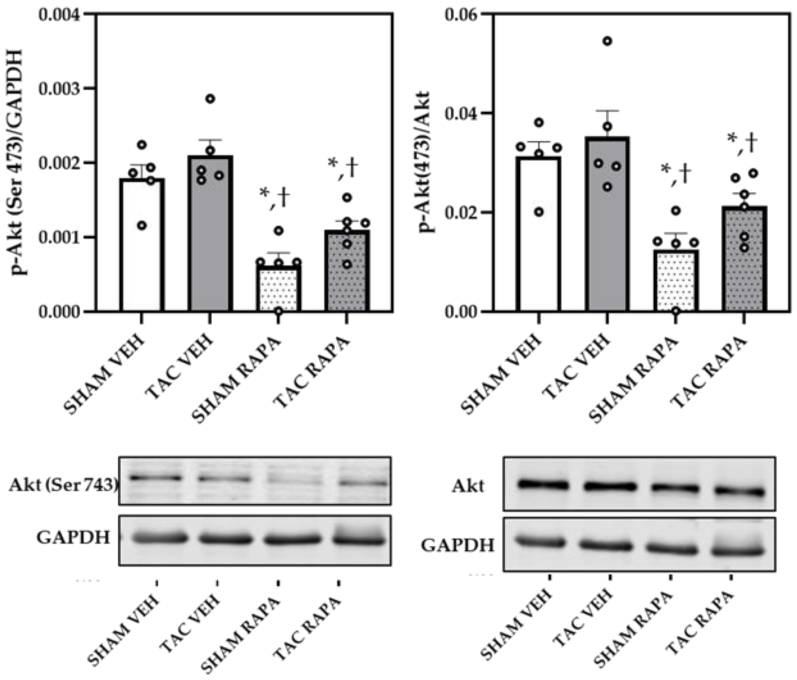

Supplemental Figure 1: Detection of Akt-Ser 473 in TAC heart tissue. Data are presented as Mean+SEM of biological replicates. Statistical analyses were performed by two-way-ANOVA followed by Tukey's. Statistically significant differences were shown by $p<0.05$, * vs. SHAM VEH and † vs. TAC VEH.

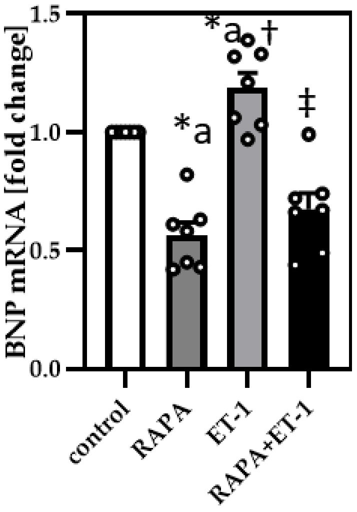

Supplemental Figure 2: BNP mRNA expression

Data are presented as fold change of control and shown as Mean+SEM of biological replicates. Statistical analyses were performed by either one-way-ANOVA followed by Tukey's posttest or one sample t-test (indicated with a), directly comparing two certain samples. Statistically significant differences were shown by p<0.05, * vs. control, † vs. RAPA and ‡ vs ET-1.
